# Understanding the implementation of the holiday activities and food programme in the North East of England using normalization process theory

**DOI:** 10.3389/fpubh.2022.954679

**Published:** 2022-09-12

**Authors:** Margaret Anne Defeyter, Tracy Finch, Eilish Samantha Crilley, Jackie Shinwell, Emily Mann

**Affiliations:** Faculty of Health and Life Sciences, Northumbria University, Newcastle upon Tyne, United Kingdom

**Keywords:** food insecurity, holiday activities and food, Normalization Process Theory (NPT), policy and institutional actions, poverty

## Abstract

Following several pilot projects, in 2020, the Department for Education (DfE) in England committed funding of £220M p. a to its Holiday Activities and Food (HAF) programme to support all 153 upper-tier local authorities, comprising City Councils, County Councils and Metropolitan Borough Councils, to provide an activity and food programme for children who are in receipt of means-tested free school meals. In this study, qualitative interviews were conducted with representatives from three Local Authorities in the North East of England who were responsible for overseeing the implementation and delivery of HAF programmes in their Local Authority area to examine how the summer HAF programme was implemented during summer 2021. Interviews were conducted with eight participants prior to the implementation of the HAF programme, and four interviews were conducted after the programme had been delivered. Using a directed content analysis approach, an interpretative framework was co-developed, based on the four constructs (and selected sub-constructs) of Normalization Process Theory. This framework guided data coding. The analysis aimed to identify and understand the barriers and opportunities in relation to HAF implementation within local authorities. Participants did not perceive HAF as a totally new initiative as many had either commissioned or delivered holiday clubs in the past. However, the increased scale and scope of HAF was perceived as highly complex, involving multiple local authority departments and stakeholders. Nonetheless, HAF funding enabled local authorities to improve the quality and reach of their holiday programmes. Strong networks and good communication between all stakeholders supported successful delivery, despite tight delivery timescales. However, the rigidity of some of the DfE guidance was a barrier for some providers, particularly the recommended delivery model of 4 h a day, 4 days a week for 4 weeks, with many individual holiday clubs struggling to meet this level of delivery, and local authority leads interpreting the guidance at a club level rather than an individual child access level. Furthermore, participants considered the HAF eligibility criteria too restrictive. Many councils were developing long-term plans for HAF delivery, integrated into planning across several departments, and all local authorities were actively seeking ways to engage with and embed HAF within local communities.

## Introduction

In the UK 14.5 million people, equivalent to a fifth of the population, live in relative poverty, after housing costs, including 4.2 million children (1). During term time, several policies are in place to support the nutritional needs of children and reduce the risk of children experiencing food insecurity and associated poor health outcomes. For example, the DfE supports Universal infant free school meals (UIFSM) to all reception, year 1 and year 2 pupils in England, means-tested free school meals, and a national breakfast club programme. Free school meals (FSM) are used as an indicator in schools for pupils from low-income households, and in England approximately one in five pupils (22.5%) are eligible for FSM (2).

However, when some of the ‘safety nets' provided by schools are absent, low-income households may experience additional financial hardship. Not all families will be food insecure during school holidays, but this risk increases in the absence of FSM, and when low-income households face additional challenges (3). Food security, the opposite of food insecurity, is defined as a condition that is met “when all people, at all times, have physical, social and economic access to sufficient, safe and nutritious food that meets their dietary needs and food preferences for an active and healthy life” (4). In addition, during the school holidays, low-income families experience the challenge of sourcing and accessing adequate and affordable childcare and play provision (5). Moreover, research investigating summer learning loss in children who live and attend schools in areas of high deprivation in the UK identified that children may lose skills and knowledge over the summer, and stagnation in learning occurs in reading and maths computation (6, 7). Teachers have also reported that poverty affects children's readiness to learn when they return to school after the holidays (8). Additionally, since the start of the COVID-19 pandemic, children have experienced disruption to their education, and have self-reported lower levels of physical activity and changes in their nutritional intake, consuming more lower nutritional quality food items when not in school, and increased social isolation (7, 9–11). Consequently, these adverse experiences can increase the risk of developmental delays and health problems in adulthood (12).

Local Authorities, rather than local government charities and religious organizations have been aware of these issues for many years and have funded hundreds of local holiday clubs, across the UK, to support families in disadvantaged communities (13). The principal aim of these clubs is to provide nutritious food and enriching activities to support families at risk of an inadequate supply of nutritious food and at risk of food insecurity (14, 15). However, provision tended to be piecemeal and fragmented rather than strategic (13, 16). Although clubs were initially viewed as a means to address holiday hunger, it soon became apparent that these clubs provide a number of notable health, wellbeing and social benefits for children and parents (17). For example, holiday clubs improved eating behaviors and reduced social isolation through the provision of positive learning experiences (14, 18–20). Additionally, attendance at holiday clubs has helped to alleviate food insecurity within the household (21). Holiday clubs address some of the challenges evident in disadvantaged communities i.e., lack of safe places for children to play, poor access to healthy food, and a lack of structured, community-based enrichment activities, and additionally, provide vital resources and signposting to services for families (22), However, the support and impact offered by holiday clubs was limited by their resources and capacity to deliver a comprehensive programme available to all children from low-income families.

The COVID-19 pandemic increased economic and health inequalities in the UK, resulting in higher levels of food insecurity and demand for food aid (9, 23, 24). Against the backdrop of the COVID-19 pandemic which saw school closures and a rise in economic hardship for low-income families, and following a series of pilot programmes, the DfE increased the funding for the Holiday Activities and Food (HAF) programme to £220M p.a., distributed across all 152 upper-tier local authorities in England (25). The aim of HAF is to deliver a programme of nutritious food and enrichment activities to all school aged children up to the age of 16 years of age who are eligible for means-tested FSM (26). However, in 2021, of the 1.7 million children in receipt of FSM, only just over 600,000 children attended HAF.

Holiday programme providers are expected to provide at least 4 days of activities and food during Easter and Christmas school holidays and 16 days of activities and food during the 6-week summer holiday period. Local authorities are responsible for coordinating the HAF programme in their local area and are encouraged to develop partnerships with voluntary and community organizations to support programme delivery. A requirement of HAF is compliance and adherence to a range of standards and policies, for example safeguarding, health and safety, school food standards etc. (25). The expectation that providers adhere to School Food Standards are the same as school term time. Whilst local authorities are expected to specifically target HAF to children eligible for FSM, 15% of HAF funding can support vulnerable and disadvantaged children who fall outside this eligibility criterion, with prior approval from DfE. Given that HAF is a new social policy, it is important to understand the implementation of HAF and evaluate the opportunities and barriers to how local authorities, organizations and individuals embed this policy within their practice and communities. This paper uses Normalization Process Theory (NPT) to provide a framework to assess the implementation of HAF at the local authority level.

### Normalization process theory

Normalization Process Theory (NPT) is a widely applied theory of implementation (27) that was developed by a multidisciplinary team of researchers, and iteratively tested and refined through empirical studies that investigated the implementation of a range of health care innovations (28). It provides a framework to explore the implementation of policies and practices within healthcare settings and how these complex interventions become embedded in health care practices (27). Its focus on how different individuals and groups work together to embed a new process into everyday practice is a key advantage of using NPT in relation to the implementation of HAF by local authorities. NPT comprises four main constructs (coherence, cognitive participation, collective action, and reflexive monitoring) which describe the different types of work that stakeholders engage in through the process of implementing and embedding a new intervention or policy. More recently NPT has been used in educational settings to identify policy learnings relating to school meals (26).

### Study aims

The aim of this study was to utilize the NPT framework to examine how HAF is currently being implemented across three local authorities in the North East of England and to use learning's from this study, highlight important opportunities and barriers, to inform and improve future HAF provision and policy.

## Materials and methods

### Design

This research study used a qualitative method to collect views of relevant stakeholders responsible for implementing HAF in local authorities in the North East of England.

### Participants and procedure

Purposive sampling was used to recruit participants from three local authorities in the North East of England to this study. The three local authorities were chosen to ensure there was a mix of both city and county and metropolitan councils, and population and cultural differences determined by local geographical boundaries, thus ensuring that different experiences of implementing and delivering HAF in the North East of England could be captured. As HAF teams within local authorities tend to be small, often consisting of four or five staff members, we invited all local authority staff directly responsible for leading and implementing HAF within each of the 3 local authorities to participate in this research. In total, 14 local authority staff were invited to participate in the research, with 8 staff consenting to take part in the research. Details of local authorities and number of participants from each local authority are presented in Table 1.

**Table 1:** Characteristics of local authorities.

**Local authority**	**% of population income deprived**	Rural/urban classification^*^	**Lead Department**	**Approach to co-ordination**	**Number of participants**
A	16.7%	Urban with major conurbation	Communities and Environment	Steering group	2
B	12.6%	Largely rural	Community wellbeing	Steering group	4
C	18.6%	Urban with city and Town	Public Health	Officers in first instance until Steering Group established	2

* Based on 2011 rural-urban classification of local authority districts in England.

### Materials

Letters of invitation, research information sheets, an opt in consent form, a demographic questionnaire and a debrief information sheet were developed for this study. A topic guide was developed to guide interviews for this study. This accorded with the four broad constructs of NPT. The questions focused on understanding and preparing for the implementation of HAF (coherence), the stakeholder's role (cognitive participation) and experience (collective action) of the implementation, reflecting on the implementation of HAF and future plans for delivery (reflexive monitoring). A copy of the topic guide is presented in Appendix A.

### Procedure

Following the receipt of ethical approval by Northumbria University's Research Ethics Committee (Ref: 33250), the letter of invitation, research information sheet and an opt in consent form were emailed to HAF leads within each local authority. HAF leads shared this information with all members of their team and potential participants were asked to contact the research team if they were interested in taking part in the research.

A total of eight participants were interviewed for this study. Interviews were conducted at two timepoints: at timepoint one (June – August) to understand implementation of HAF at the early stage of embedding the policy, and timepoint two (September – October) following the delivery of HAF in the school summer holidays. All participants were invited interviewed at timepoint one, on a one-to-one basis. At timepoint two, four HAF programme leads participated in a follow up interview. Due to time pressures and at their request, two participants from the same local authority were interviewed at the same time.

Participants provided written, opt in consent. At the time of data collection, COVID-19 lockdown restrictions were in place and Northumbria University ethics procedures prohibited face-to-face data collection. Hence, all interviews were conducted on-line *via* Microsoft Teams. A date and time for each interview was agreed, and an email with a link to an on-line meeting was sent to each participant. At the start of each interview, participants were asked to verbally confirm that they were happy for the interview to be recorded. At the end of each interview, each participant was thanked for their participation and was sent a debrief information sheet. Interviews lasted an average of 48 min, ranging between 30 and 70 min. All interviews were audio recorded and a verbatim transcript of each interview was produced.

### Data analysis

Directed content analysis (29), a deductive, framework-driven approach, was undertaken to code the data in relation to the constructs of NPT. In its theoretical specification (27), the constructs and propositions that underpin NPT are framed generically. For application in specific projects, the authors advise (30) researchers to translate the constructs into project-specific statements and questions to guide data collection and analysis. This approach was chosen to enable us to achieve a direct exploration of barriers and facilitators to HAF implementation, from the perspective of NPT. This approach was used successfully to describe NPT related barriers and facilitators to implementing a new assessment tool in Swedish psychiatry provision (31). Given the deductive nature of this approach, the research team co-developed the interpretative NPT framework (Table 2), based on the work of Gunn et al. (32), to ensure appropriate translation of the NPT constructs into working definitions consistent with the HAF programme setting, whilst retaining strong alignment with the theoretical constructs of NPT. To enable additional depth of exploration of the data in terms of ‘how HAF implementation happened in practice', we developed the ‘collective action' construct into its more detailed components (interactional workability, relational integration, skillset workability and contextual integration). These sub-components of collective action, in combination, represent the original predecessor model to NPT, Normalization Process Model (33), and is the construct most tested in other NPT framed research (34).

**Table 2:** An interpretative framework of NPT developed and applied for analysis based on the work of Gunn et al. (32).

**Propositions developed and explored**	**NPT construct**	**Our interpretation of construct to guide analysis**
Implementation of HAF requires conceptualization	Coherence: Do people understand what HAF is? What are the key elements of the intervention?	Is HAF viewed as a new social policy? What work has been undertaken to reach a shared understanding of the policy aims and outcomes? What are the stakeholders understanding of the benefits of engaging with the policy?
Implementation of HAF requires engagement of a range of stakeholders	Cognitive participation: Which stakeholders are engaged and involved in implementing and embedding HAF?	How do stakeholders engage with implementing and embedding HAF in their local authority? What are the practices required to sustain HAF? What work is undertaken to ensure stakeholders recognize their role in policy implementation?
Implementation of HAF requires agreement about how HAF is implemented, delivered and sustained	Collective action: Interactional workability and skill-set workability	Interactional workability: How is the implementation of HAF conducted? What range of interactions did stakeholders encounter that enabled or hindered tasks?
		Skillset workability: Is the work allocated to appropriately skilled staff to implement HAF?
		Relational integration: Do stakeholders have confidence in new practices to sustain the implementation of HAF?
		Contextual integration: Is the implementation of HAF shaped by resources and policies available?
Implementation and delivery of HAF requires ongoing assessment of how the programme is delivered across the region	Reflexive monitoring: Appraisal of the policy	How do stakeholders review and reflect upon the implementation of HAF? How is HAF monitored within local authorities?

Transcripts were the main unit of analysis and were read several times prior to coding, to develop familiarity with the data within and across transcripts. Transcripts were uploaded into Nvivo 12 for ease of access.

## Results

The results of our analysis are presented under the four main NPT constructs including quotes from participants to demonstrate each theme. Each participant was allocated a number, and each quote is followed by a number in order to indicate its source.

### Coherence

The first construct explores whether HAF is viewed as a different way of working within local authorities. No participants described HAF as a completely new way of working. Whilst only one local authority in this study had been awarded HAF pilot funding in previous years, all participants, or the local authority they represented, had previously commissioned, implemented or delivered a programme of holiday activities. Prior to HAF, local authorities had developed networks and relationships with a range of delivery partners within their region and models of provision had evolved and developed. However, the DfE guidelines on the type of activities offered and food delivered through the HAF programme may differ from previous programmes of holiday provision delivered by local authorities. For example, the DfE requires the inclusion of a nutritional educational element to be incorporated in the programme of activities, and all delivery sites to provide food that meets school food standards, and the availability of provision for children with SEND. Additionally, a requirement of the DfE is for HAF to be available for all means-tested FSM children within a local authority and hence, several existing models needed to be significantly upscaled in terms of capacity, reach, and participation criteria. To provide evidence of need and provision, a criterion of HAF funding is for local authorities to map need against HAF provision. Thus, HAF differs from the rather piecemeal implementation of previous holiday club schemes as demonstrated by the process of detailed mapping and analysis to identify and plan HAF delivery locations:

“We have a map of [name of region] which includes postcode data of all children eligible for free school meals. So we can look at any part of that map and say well actually that is where our children on that benefit live. On top of that we can put all sorts of layers, so we can put schools we can put community facilities, we can put our existing providers and partners, we can do some proximity mapping so we can work out how close to a particular location and programme of activity our children live and how far they have to travel either by vehicle or walking” (Participant 5).

Participants recognized that there were differences between, and within, local authorities (in terms of demographics, rural and urban areas, and resources) so that there is not a ‘one size fits all' approach to implementing and delivering HAF. It was recognized that HAF encompasses many local authority departments and services and the responsibility of which department HAF should be under rests with each local authority. One participant noted that through HAF funding, they were able to commission a HAF lead to deliver the programme of activities across their region.

A requirement of DfE funding for HAF is the formation of a steering group. Participants described how steering groups represented the broad views from across each local authority and comprised a mix of council services, key partners from private sector, community organizations, and charity providers. In addition, some participants sought views from school leaders, representatives from the Youth Council, and local community food networks. Nevertheless, one participant discussed that due to the restricted timescale, the need to make rapid decisions, the changing landscape with regards COVID restrictions and guidance, as well as conflict among stakeholders with regard to the commissioning process, they decided not to establish a steering group during the initial stage.

“Some of that probably should have gone through what we were supposed to set up which was a holiday activities steering group or something like that. Actually, at the end of the day I didn't set that group up because one I didn't want to take any more abuse, and two the programme was changing so dramatically that the steering group could have made the recommendation on the Monday and by the Tuesday it could have changed” (Participant 1).

Additionally, one participant highlighted the challenge of the additional time and resources needed to implement these steering groups:

“I don't think everybody was prepared for the amount of time this would take and the amount of effort it would take” (Participant 6).

Participants highlighted that through discussions with stakeholders from a range of organizations, they identified a greater understanding of the challenges and needs of families during the school holidays, and many needs were beyond the scope of supporting families claiming FSM (e.g., children with education or care plans, children living in poverty but not eligible for means tested FSM, and safeguarding children at risk of domestic abuse). Participants expressed frustration with the limited scope of HAF and rigid guidelines of the policy and described that by focusing solely on supporting children eligible for means tested FSM, they miss many vulnerable children including low-income families that fail to meet the eligibility threshold for FSM:

“there is still the issue there of families that are above the threshold for help with benefits, they don't get free school meals they sometimes struggle more so than the families who are in receipt of benefits” (Participant 5).

They expressed a desire to develop HAF to help families in need across their region, regardless of FSM eligibility. Participants, with support from steering group members, developed a set of broader objectives, beyond the scope of HAF and the means tested free school meal eligibility criteria. By securing funding through other funding streams, they were able to deliver HAF to a range of families in need of support:

“when the DfE funding was announced we realized that there was- I think it was around seven thousand children who are eligible for free school meals but there's also seven thousand that were experiencing poverty who would need to- who would really benefit from accessing but they don't get free school meals so wouldn't be able to under HAF, so that's why we applied for funding under the lottery to be able to do this” (Participant 4).

### Cognitive participation

Participants discussed how they worked hard to develop a good working relationship with DfE and received good support from the DfE in terms of implementing HAF. Nevertheless, participants identified that communication from DfE could be improved to facilitate the delivery of this provision. Most participants described that a core team was created within the local authority who were responsible for the day-to-day tasks of implementing HAF, and this group met with a steering group or coordinating group on a regular basis. Steering or coordinating groups provided guidance and advice in overcoming specific issues for example, food provision and risk assessments. Whilst the responsibility for overseeing the implementation of HAF rested with one or two individuals, the formation of a small working team and wider steering group were important elements in delivering HAF.

“I would say the steering group have a really active role in helping us to shape the thinking round the programme. It doesn't have any formal decision-making responsibility, but I mean if the people around that table told me that something was a bad idea or a good idea then generally speaking, we try and run with it I guess ultimately the decision lie with me” (Participant 5).

Another participant similarly recalled that they ultimately accepted full responsibility for the implementation of the HAF programme in their local authority area. This was partly because the rate at which policy directives from the DfE were changed, that a decision made 1 day by a steering or coordinating group based on guidance from the DfE could be out of date the next. As a result, to facilitate co-ordination, the role and name of the steering group was changed to a co-ordination group in one local authority:

“…the programme was changing so dramatically that the steering group could have made the recommendation on the Monday and by the Tuesday it could have changed. So I just yeah it's not (interruption) so we just we just had that group meeting this morning for the first time and I changed the name of it from holiday hunger steering group to holiday hunger coordination group to try and coordinate all of the activities across the board” (Participant 1).

The majority of participants reflected that building relationships is a vital aspect of ensuring commitment and engagement from a range of stakeholders. One participant described how each member of the core working group had developed a range of relationships with different sectors (e.g., schools, voluntary and community sector) which helped with the process of implementing the policy. Whilst many organizations were keen to be involved in the delivery of HAF, the COVID-19 pandemic resulted in a mixed reaction from delivery partners. Some voluntary and community organizations were unable to commit to the programme as closures during lockdown had left them unprepared and under-resourced to deliver HAF. Participants recognized a similar mixed response amongst schools: in some areas schools did not want to engage with the programme as teachers were concerned about the workload for staff and not wanting to open up schools, whilst other schools viewed this programme as an opportunity to reengage with families. In addition, one participant reflected that the lack of youth providers in their region proved a challenge to delivering a programme relevant for adolescents:

“There just isn't a mature market of youth provision in [name of region] because it's still kept in house in the council so there isn't an army of VCS organizations that aimed at providing youth services in [name of region] because it's in house” (Participant 8).

Participants reflected that planning and delivering HAF is complex, particularly ensuring the quality of provision for SEND children. Participants reflected on the detailed planning and documentation of evidence to ensure the quality of activities delivered and safeguarding of children:

“We do a lot of preparation which we have spoken about in terms of our planning. We have lots of documentation which you know ensures our activities are safe and of a high standard” (Participant 5).

One participant described how they were developing a framework to clearly set out the responsibilities of those involved with implementing HAF, both within the local authority and external delivery partners:

“It is a massive job I didn't quite realize how much work was involved in it, but I think now that [delivery partner] is involved in it I think that we will be able to start to develop a framework on how we are going to deliver. That is my next step with [delivery partner] to look at that framework and look at the kind of different steps that we need to go through and what is the expectation and what is our role because I do feel that I probably need to bring a bit more back into the local authority” (Participant 2).

An issue with the implementation of HAF at the local authority level is that it sits across service departments and different departments have taken the lead for HAF within different local authorities. Regardless of which service department was responsible for leading on the delivery of HAF, delivery leads described their role as a supportive, coordinating role: developing intelligence and understanding the needs of the community; and building relationships and links with a range of delivery partners to deliver a programme of activities:

“So, I tended to be quite a link between the schools and the HAF programme. So, it's all about the relationships you've already got with head teachers and staff. A lot of the things I suppose it's the intelligence that you've got around the education and what's already happening in schools so that that could be, I suppose, used, expanded as part of the HAF programme, opening doors for the voluntary sector to be able to get obviously use of buildings, etc. … So, I think that's probably a lot of what I've brought to the group. I said my role has very much just been a supportive one” (Participant 7).

### Collective action

#### Interactional workability and skillset workability

The sub-constructs of interactional workability and skillset workability are discussed together. Participants described relationships with stakeholders and highlighted actions that enabled or hindered tasks. The majority of participants referred to communication as a vital component for building relationships, to ensure tasks are achieved and to overcome barriers. One stakeholder implementing and delivering HAF described how they had frequent team meetings to ensure all tasks were achieved and members adhered to an agreed timescale:

“In terms of that sort of preparation just really good communication, regular team meetings built in and having to just say right this is a cut-off date now. So I have kind of said in today's meeting, Wednesday is our final cut off because we have got people who are going to get back to you next week, well they haven't so it's like okay (says name) I need you to ring and that's it Wednesday's cut off and we will go with what the next steps are for the programme” (Participant 2).

Participants highlighted how each member of the team had a different set of skills, experience and networks that were necessary to ensure all elements of delivering HAF were covered.

In addition, building and managing relationships with stakeholders were important to enabling the delivery of HAF. Developing relationships with senior stakeholders from a range of different sectors (e.g., voluntary and community sector, private sector, and public sector) enabled HAF leads to help build capacity for delivery partners, link delivery partners to a range of organizations and support, and ensure that a wide range of tasks were achieved on time. Participants described how the development of relationships with a range of organizations enabled them to develop not only a comprehensive programme of activities but also contingency plans that could be implemented if the programme was affected by the COVID-19 pandemic restrictions or adverse weather. Key tasks were delegated to different stakeholders and sectors e.g., the local authority catering team and trading standards team were consulted on the provision of food, and youth leaders and volunteer leads supported recruitment of volunteers:

“They had already had their plans they knew what was happening they knew what the food offer was going to be like. It was about opening that up to the kind of wider community and what we could do and what we couldn't do” (Participant 2).

Nevertheless, some participants reflected on the challenge of delivering HAF in accordance with the DfE guidelines, limitations of what delivery partners could either provide or unwillingness of delivery partners to meet these guidelines. Participants described the challenge for some delivery partners to deliver hot food, and to provide, as a minimum, the 4: 4: 4 model of delivery (4 h per day, 4 days per week, for 4 weeks of the school summer holidays):

“I have been given a national specification to deliver on and that is what I am trying to deliver on, my providers are saying can't do the hot meals, can't do that so we have sorted those ones, but then they are saying they can't do the 4,4,4, it's just too much as our staff want to take holidays or we are not going to get the kids turning up for that amount of time …and I am just like tearing my hair out” (Participant 1).

Conversely, one external delivery partner reflected on the challenge of working with local authorities and were critical of how the local authority allocated DfE funding:

“I would say 25 per cent of the local authorities we have worked with have taken what I call a top slice, so a percentage of the funding off which has made it impossible for us to deliver. So about 25% of the projects we have looked at or been invited to look at we have said no to, because it wasn't financially viable to do so, mostly because the money the local authorities top sliced has made it prohibitive. That's happened in quite a few cases I would imagine that local authorities would also want to ringfence some of the money to cover some staff costs and to put into their own budgets for managing and overseeing the project” (Participant 3).

#### Relational integration and contextual integration

Participants did not describe in detail the integration of practices and thus, the sub-constructs of relational integration and contextual integration are discussed together. Participants with previous experience of implementing and delivering HAF, have developed a long-term working plan of delivering a programme of activities and have built this into their practices:

“It's built into our work now and we are a team that is comfortable and familiar with this way of working. There is lots of areas that have brought in coordinators to deliver this year's programme, many of whom have no experience of this area of work so you know I think that a lot of them are doing absolutely fantastic stuff and there is also lots of learning that we have discussed that a lot of them can use from elsewhere” (Participant 5).

Several key issues were discussed by participants relating to how work was shaped by resources and policies. All participants mentioned that the COVID-19 pandemic had affected the implementation and delivery of HAF, and they developed contingency plans in the event of no face-to-face provision. Participants reflected that the COVID-19 pandemic had also made them consider alternative ways of providing activities:

“COVID gave us some great opportunities to do things differently. Some of our remote stuff as well you know we got support, not ideal because you know I am a big advocate of face to face but we got support to some children and families that we have never engaged in a holiday club previously. That's really interesting that it has engaged extra people from the online format” (Participant 5).

### Reflexive monitoring

Participants reflected on the formal and informal collection of information and data to evaluate the efficacy of the programme. As a requirement by the DfE, participants collected data on attendance and FSM status of attendees. In addition, the majority of participants described an informal process of evaluating HAF. Participants asked for reflections from delivery partners and parents as well as monitoring Facebook posts to gauge the impact of the provision in the community. However, many participants reflected that they were developing a more systemized approach to capture data. One participant highlighted that the local authority is changing their approach to monitoring programmes and no longer evaluating programmes in isolation but in connection with a range of other programmes delivered across council departments e.g., youth work, leisure contracts, emergency food aid. Thus, monitoring how the outcomes of one programme impact on how other programmes are delivered:

“So rather than saying, how was the HAF, it's kind of about, you know, we did our school uniform grant differently, we've got our COVID support grant, and we are doing that differently, the welfare assistance, we are doing differently with families, all sorts of emergency food we are doing differently so it's about how is this all going and it's in that mix. It's not a standalone programme, it's part of a wider approach to getting it right earlier, in a bigger way” (Participant 6).

In one borough, staff from the local authority visited each venue at the start of the holidays to ensure the programme of activity adhered to DfE's requirements including type and quality of food delivered and safeguarding procedures:

“One of the things I haven't mentioned but we do make sure, we do have somebody on site at the commencement of every activity, not every single day but when a holiday club starts up. On the 1st day of its activity whether it be day one of week one or day one of week three, there will be somebody from the team that will visit that holiday club or provider to make sure that they are adhering to everything that they should be” (Participant 5).

Participants reflected that a key element of delivering a comprehensive HAF programme is building relationships and trust within the community. The development of relationships with delivery partners, community organizations and parents requires time. Whilst building relationships was mentioned by the majority of participants, a key challenge was the very tight timescales. The lack of time meant that it proved difficult to build trusting relationships with delivery partners as well as for stakeholders to contribute and work collectively. Therefore, sufficient timescales are required to develop relationships and help increase the scale, capacity, and quality of HAF across the North East of England:

“It comes back to the timescales, the longer we have to prepare, to market to build relationships and networks the more effective this programme is going to be” (Participant 3).

Participants reflected on the impact of HAF on children and young people as well as their parents. A key benefit for children and young people attending HAF was social contact, particularly following periods of lockdowns and isolation. In some regions, young people were upskilled to become young leaders to support the delivery of the programme or young ‘inspectors' to help evaluate the programmes:

“We're doing with this young leaders' programme, and I think that will have a long-lasting legacy, really. Young people organizing activities for the for the young people themselves. So, I think it's I think it's really, really beneficial. And I think that it's growing future leaders” (Participant 7).

Participants viewed that the DfE guidelines, together with increased HAF funding, enabled them to improve the quality of the programme of activities provided. One participant for example recalled how the extra funding from HAF had meant they had been able to add and embed good quality food provision into their programme. Another participant suggested that the ability to target support at vulnerable young people had been particularly effective:

“but I think the actual programme itself and the targeted support it's really effective… it's been about targeted support for some of our more challenging young people. Shall we say, those that are probably at risk of permanent exclusion” (Participant 7).

By developing relationships and trust with families, community organizations and delivery partners, participants were able to identify gaps in provision and endeavor to support organizations to fill these gaps. In addition, one participant described how they are seeking to engage with parents and children to co-produce future programmes to ensure that the service users have an active voice in shaping the HAF programme within their local authority.

“The next plan for the programme of activities based in [name of town] will be on the voices of children and their parents and that it is a co-produced programme … It needs to be built into the programme that you have evidence and demonstrate how you engage with your residents to enable that programme to be coproduced on the needs and views of children and mums and dads” (Participant 5).

The majority of participants reflected that HAF provided them with multiple learning opportunities. Whilst they had experienced a range of challenges in terms of implementing and delivering a complex programme of activities, including responding to COVID, participants positively reflected on the learning opportunities and the ability to think around challenges. Nevertheless, for several participants, the workload of implementing HAF was stressful particularly as this was in addition to their current role:

“The number of sleepless nights this has given me. I mean just for an example, last Saturday morning I woke at 2 in the morning in a panic that I had completely forgotten the Ofsted requirement fee … it is all consuming” (Participant, 8).

Despite the challenges and additional workload, one participant reflected on the pride of delivering a complex programme of holiday activities and food:

“I'm really proud of the diverse range of activities that we have on our programme. I think it has gone as well as I could have wanted it to under the under the limitations of COVID I think as I said I am really comfortable that our programme is good it's broad” (Participant 5).

## Discussion

In our opinion NPT provides a useful framework to examine how HAF is implemented by local authorities and provides insights into key factors affecting how HAF was implemented, with reference to coherence of the policy, how individuals and organizations embed and implement the policy, the role of collective action, and appraisal of implementing HAF.

In relation to coherence and understanding HAF, all participants had prior experience of delivering some form of holiday programme. One local authority had participated in the Department for Education's HAF pilot projects and this funding had enabled the local authority to further develop extensive social networks and relationships across the community, voluntary and private sectors, the DfE, and a more comprehensive understanding of how the HAF programme is delivered across their region.

Local authorities are required to comply with the DfE's HAF guidelines, including mapping of need against HAF providers, delivering HAF to children eligible for means-tested FSM, and delivering a model of provision that comprises 4 h of activities and food a day, 4 days a week, for 4 weeks of the school summer holidays (4:4:4 model), school food standards, physical activity guidelines etc. Although participants recognized the need for accountability and quality assurance, all participants expressed their frustration with the limitation of the current HAF eligibility criteria and the lack of flexibility with certain elements of the policy. There was a consensus among participants relating to the limitation of HAF only supporting children eligible for means-tested FSM. Participants recognized a need to extend the scope of this provision to reach vulnerable families beyond FSM criteria and to reduce stigmatization of the programme. Prior evidence suggests using FSM as a proxy measure for poverty does not accurately measure the number of children from low income or food insecure homes, particularly in light of a rise in families experiencing food insecurity since the start of COVID-19 pandemic (34). Moreover, an evaluation commissioned by the DfE showed that only 600,000 children in receipt of FSM attended the HAF programme in 2021, representing a small proportion of the 1.7 million children eligible for free school meals, and a smaller proportion of the estimated 3.9 million children in poverty. Participants in the current study suggested that removing the FSM eligibility criterion, allowing local authorities to have discretion to target provision at the ward and/or neighborhood level rather than the individual level would result in a more inclusive, less stigmatizing, and easier to administer programme that would drive attendance. Alternatively, the DfE could extend the programme to include all children in need, regardless of FSM status.

In addition, participants reflected that the 4:4:4 model of delivery created a barrier for some local providers to engage in the HAF programme. Many local organizations were unable to deliver this model due to resource issues, specifically in terms of recruiting staff and volunteers. Although parents and carers often request holiday clubs to open more frequently and for longer hours across the school holidays (17), without additional resources it seems that local authorities and local organizations are unable to meet parent's needs.

To develop a collective understanding of the principles and objectives of HAF within their region, participants within two local authorities established steering groups and sought views from a range of stakeholders from across local authority departments, school leads, private sector, community and voluntary sector, community food networks and Youth Council. The establishment of steering groups helped local authority staff to develop an understanding of need for HAF, develop partnerships and networks which supported the implementation phase. Nevertheless, the lack of time to plan and implement was a continuing theme throughout implementation phase of HAF and the tight timescales prevented one programme lead from establishing a steering group during this initial phase. However, this local authority realized the need for a greater co-ordination and set up a coordinating group during the delivery phase of their HAF programme.

Participants reflected on their engagement with the policy regarding HAF, what was expected of them and how the different responsibilities fitted together to drive forward the implementation of HAF. Within two local authorities, a small working committee was developed of several individuals that had prior experience in delivering and supporting the different areas of HAF (e.g., physical activities, food, marketing etc.). The development of a framework to communicate responsibilities helped to ensure that the extensive range of tasks were assigned and actioned by individuals e.g., coordination of delivery partners, compliance and safeguarding procedures, building and developing relationships with community, voluntary and private sector. Regular communication amongst working committee members helped to address issues as they arose and ensured agreed timescales were met. Conversely, in the absence of a working committee, programme leads experienced increased workload and pressure to implement and embed HAF without additional support and resources. In relation to collective action, a barrier for some participants in embedding HAF into their working practice was managing relationships and expectations across local authority departments as well as the community, voluntary and private sectors. Again, additional time will help to forge these relationships, build trust, and embed HAF within local authorities and communities.

The HAF programme was extended to include all 152 upper-tier local authorities in 2021 during the COVID-19 pandemic. This resulted in additional challenges. Against the backdrop of implementing and embedding HAF, rules and guidelines to manage the spread of COVID-19 were regularly changing, including national and regional lockdowns, rules governing self-isolation with COVID-19 or a close contact, as well as social distancing (35). As a result of the Covid-19 pandemic, there was a need to develop contingency plans, in addition to a comprehensive programme of enrichment activities, food provision and nutritional education, in the event that face-to-face provision was unable to take place. Moreover, it is evident that schools were less willing to engage with supporting the delivery of HAF, compared to community organizations. The lack of cooperation may, in part, be a result of successive school closures and changes to teaching arrangements since the start of the pandemic in 2020 impacting the health and wellbeing of staff (36). In addition, schools may have been cautious about the potential of needing ‘deep cleaning' to be carried out due to COVID. Regardless, we encourage the DfE, local authorities and community organizations to collectively seek solutions to this matter, perhaps considering funding for opening up schools to community organizations to deliver HAF across the school holidays.

Finally, participants responsible for embedding HAF within the local authority have gathered formal and informal evidence to evaluate the implementation of HAF. Examples of this evidence includes regular site visits to holiday clubs by local authority inspectors to ensure delivery partners are complying with HAF criteria and guidelines and to identify good practice. To help embed HAF in local communities, participants described how they used other interventions and programmes to help support the delivery of HAF e.g., youth leader programmes, the violence reduction programme etc. In addition, to help improve the reach of HAF, a number of local authorities have plans to engage with the wider community including, young people and families, to co-design future HAF programmes to ensure the programme meets and addresses local needs.

### Recommendations

We propose a number of recommendations to the DfE and to local authorities. First, we suggest that the DfE guidance on the 4:4:4 should be made clearer, ensuring that local authorities are aware that this model is the minimum recommended level of individual child access to the programme, and that this may be achieved by local delivery organizations working in partnership to ensure all children are able to access 4 h a day, 4 days a week, for 4 weeks of the year as a minimum from across a number of local venues. Second, we propose that the eligibility criteria for HAF be reconsidered to (i) reduce stigmatization of the programme, (ii) facilitate uptake and attendance, (iii) and to ease the administrative burden of the programme at the local level. Third, we propose that the DfE supports local organizations to enable them to register with Ofsted, thus providing parents with the opportunity to use their working child tax credits to further increase access to quality childcare provision across the school holidays, whilst providing additional financial resources to local delivery organizations to meet demand. Fourth, we recommend that local authorities, establish a steering group and a working committee to support HAF programme development in terms of service user's experiences and specialized knowledge (e.g., School Food Standards etc.). Finally, we recommend that local authorities evaluate and monitor implementation and development of the HAF programme at the local level using the NPT framework.

This study is the first study to investigate the implementation of HAF in three local authorities across North East of England using a NPT framework. Findings from this study highlight important opportunities and barriers of implementing, embedding, and sustaining HAF at the local level. The use of a robust, theoretical framework to frame data collection and analysis is a strength of the study, as it allowed us to undertake a detailed and comprehensive investigation of factors affecting the implementation of HAF. It is possible however, that other theories and approaches may have led to identification of a different set of influencing factors. Using a deductive approach to data analysis, may thus have some limitations in the extent to which other explanations for the implementation of HAF may have been developed in our study. In 2012, there were reportedly 61 frameworks for implementation and dissemination research (37), and the field continues to develop. Choice of framework is therefore critical. NPT pays particular attention to mechanisms of implementation, where ongoing collaborative work involving multiple stakeholders and participants is key to successful implementation. This aligned well with our understanding of the HAF programme setting, and we suggest it served our purpose well of identifying and understanding factors affecting the implementation of HAF. We hope that earnings f from this study will help inform the development and expansion of this important social programme.

## Data availability statement

The raw data supporting the conclusions of this article will be made available by the authors, without undue reservation.

## Ethics statement

The studies involving human participants were reviewed and approved by Faculty of Health and Life Sciences, Northumbria University. The patients/participants provided their written informed consent to participate in this study.

## Author contributions

Conceptualization and funding: MD. Methodology: MD and TF. Formal analysis: EM. Data curation: EM, EC, and JS. Writing original draft preparation: EM, MD, TF, and EC. Writing, reviewing, and editing: EM, MD, TF, EC, and JS. Project administration: EM and MD. All authors have read and agreed to the published version of the manuscript.

## Funding

This project was jointly funded by Gateshead Council, Northumberland Council, and Middlesbrough Council.

## Conflict of interest

The authors declare that the research was conducted in the absence of any commercial or financial relationships that could be construed as a potential conflict of interest.

## Publisher's note

All claims expressed in this article are solely those of the authors and do not necessarily represent those of their affiliated organizations, or those of the publisher, the editors and the reviewers. Any product that may be evaluated in this article, or claim that may be made by its manufacturer, is not guaranteed or endorsed by the publisher.
