# Supplementary material for: Understanding the implementation of the holiday activities and food programme in the North East of England using normalization process theory
Source: Front Public Health. 2022 Sep 12;10:954679. doi: 10.3389/fpubh.2022.954679 (PMC9510688; doi:10.3389/fpubh.2022.954679)
Supplement: Supplementary file 1 [file Data_Sheet_1.docx]

Appendix A

Topic guide for interviews

Coherence questions

1. How would you describe the HAF programme?

2. Can you tell me about your job role and why you got involved in the implementation of the HAF programme? (Prompt: Did you clearly understand your role within the implementation of the HAF programme? How were you informed of the HAF programme?)

**Covid related question:**

**3. What was your role in relation to ensuring that holiday provision was Covid compliant?**

4. How do you feel that HAF can potentially benefit service users? (Prompt: have your assessments of this changed at all since you got involved? If so, how?)

Cognitive Participation questions
1. What preparation was needed to implement HAF? (Prompts: was the time commitment and effort which was needed, as you anticipated?)

**Covid related question:**

**2. Were you prepared for the impact that the Covid pandemic may have had on your holiday provision plans/implementation?**
3. Who was driving this forward/encouraging involvement in the HAF programme in your local area?

4. How are the decisions made about how HAF is implemented in your local area? (prompts: who makes those? Who contributes to this process?)

Collective action questions
1. What did you find easy and what did you find difficult (i.e., the main challenges and barriers) about implementing the HAF holiday programme? (Prompt: Have you adopted any strategies to overcome any of these?)

2. In your opinion, what do you think are the main important elements for successfully implementing the HAF programme?

3. Can you talk about the training/support you received from the outset to implement the HAF holiday programme, if any? This could be within your local authority of led by national government (Prompt: Was this helpful/unhelpful and if so, in what ways?)

**Covid related question:**

**4. Were you given any specific training, advice or guidance relating to Covid?**

5. Do you have a protocol or certain procedures that you follow to implement the programme? (Prompt: What are the protocols/procedures that you follow?)

6. How did you need to work with others to implement the HAF programme? Who did you work with and what was that experience like?

**Covid related question:**

**7. Did you have to work with others to make sure that the clubs were/will be Covid compliant?**
8. How do other organisations and/or programmes in your area impact on your implementation of HAF? (Prompt: If any, are these helpful or not?)

Reflexive monitoring questions

1. How do you feel the implementation of HAF has gone? Prompt: is this how you might have expected? (why/why not/ in what ways?)

**Covid related questions:**

**2. What impact do you think the Covid pandemic has had on the implementation of the HAF programme, and the holiday provision sites?**

3. Have you been able to make any judgements about the HAF programme so far? (prompts: What about in terms of appropriateness? What about in terms of effectiveness? What sources of information have informed these assessments? If so, how and what did you find?)

4. What feedback have you come across, from service users who attended HAF?

5. Have you adapted your delivery of HAF since it started? If so, how, what prompted that?

6. How might the implementation of HAF holiday programme be improved?
7. How sustainable do you think the HAF programme is in the long term?

Is there anything that you would like to mention or add that you do not think we have covered?
